# Human vs. Mouse Nociceptors – Similarities and Differences

**DOI:** 10.1016/j.neuroscience.2017.11.047

**Published:** 2018-09-01

**Authors:** Charlotte Rostock, Katrin Schrenk-Siemens, Jörg Pohle, Jan Siemens

**Affiliations:** a Department of Pharmacology, University of Heidelberg, Im Neuenheimer Feld 366, 69120 Heidelberg, Germany

**Keywords:** CIP, congenital insensitivity to pain, DRGs, dorsal root ganglia, GDNF, Glial-Derived Neurotrophic Factor, IEM, inherited primary erythromelalgia, LTMRs, low threshold mechanoreceptors, PEPD, paroxysmal extreme pain disorder, ROI, regions of interest, SFN, small fiber neuropathies, TH, Tyrosine hydroxylase, TRP, Transient Receptor Potential, VGSCs, voltage-gated sodium channels, sensory neurons, dorsal root ganglia, marker expression, human nociceptors, TRP ion channels, chronic pain

## Abstract

The somatosensory system allows us to detect a diverse range of physical and chemical stimuli including noxious ones, which can initiate protective reflexes to prevent tissue damage. However, the sensation of pain can – under pathological circumstances – outlive its usefulness and perpetrate ongoing suffering. Rodent model systems have been tremendously useful to help understand basic mechanisms of pain perception. Unfortunately, the translation of this knowledge into novel therapies has been challenging.

We have investigated similarities and differences of human and mouse peptidergic (TRKA expressing) nociceptors using dual-color fluorescence *in situ* hybridization of dorsal root ganglia. By comparing the transcripts of a selected group of well-established nociceptive markers, we observed significant differences for some of them. We found co-expression of *Trpv1*, a key player for sensitization and inflammatory pain, with *TrkA* in a larger population in humans compared to mice. Similar results could be obtained for Na_v_1.8 and Na_v_1.9, two voltage gated sodium channels implicated in pathological forms of pain. Additionally, co-expression of *Ret* and *TrkA* was also found to be more abundant in human neurons. Moreover, the neurofilament heavy polypeptide was detected in all human sensory DRG neurons compared to a more selective expression pattern observed in rodents.

To our knowledge, this is the first time that such detailed comparative analysis has been performed and we believe that our findings will direct future experimentation geared to understand the difficulties we face in translating findings from rodent models to humans.

## Highlights

• Comparative analysis of TrkA expressing nociceptors in human versus mouse dorsal rot ganglia.
• Double fluorescence *in situ* hybridization to assess similarities and differences in sensory neurons of the two species.
• The fractional abundance of TrkA positive neurons co-expressing specific receptors is different in rodents and humans.
• Results have implications for translating data from rodent pain models to human pain pathologies.

## Introduction

For many decades researchers have used lower vertebrates to study somatosensation and pain. Model organisms, in particular the mouse, have served as invaluable tools to help understand basic signaling pathways, neuronal networks and molecular aspects involved in the transduction of sensory stimuli resulting in the perception of pain (Mogil, 2009). With the development of more sophisticated genetic tools (such as intersectional genetics (Madisen et al., 2015)), it is now possible to focus on molecularly-defined neuronal populations *in vivo* with the goal to dissect their role in specific pathological pain states in rodents. In light of these more refined methodologies and given the observed discrepancy of rodent and human pain phenotypes (Hill, 2000, Percie du Sert and Rice, 2014, Burma et al., 2016), it becomes ever more important to understand the differences and similarities between animal models and human physiology.

The call for translational approaches has become louder over the past years with researchers, pharmaceutical companies and reviewers from funding agencies envisioning that this could help accelerate drug development and finding potent compounds for pain-impaired patients. In many medically related areas it appears by now to be the norm to use human subjects, tissues or cell models in order for a study to be accepted by the scientific community to be of relevance. But while it might be easier to obtain human tissue to study diseases such as cancer, the peripheral nervous system is not easily accessible. In particular, the dorsal root ganglia (DRGs) as well as the trigeminal ganglia, two anatomical sites containing the majority of primary sensory neurons, are difficult or even impossible to access in a living human being and even proof complicated to obtain postmortem.

The discovery of human induced pluripotent stem cells and the (limited but possible) access to human embryonic stem cells offers yet another possibility to work with human material by differentiating them into human sensory neuron-like cells (Chambers et al., 2012, Blanchard et al., 2015, Schrenk-Siemens et al., 2015, Wainger et al., 2015). But again, such a cellular approach is also faced with the question how similar the *in vitro* generated neurons are compared to their native human counterparts, a problem that cannot be easily addressed because most previous molecular analyses of nociceptive neurons have been performed using rodent – but not human-tissue (Reinhold et al., 2015, Reynders et al., 2015, Usoskin et al., 2015, Li et al., 2016).

In an attempt to compare the status quo of nociceptive sensory neuron marker expression in human versus mouse DRGs, we acquired postmortem human DRGs of human donors from the Netherlands Brain Bank (www.brainbank.nl).

Throughout the study we used human DRG tissue obtained from five unrelated individuals to account for inter-individual differences. We compared the combinatorial co-expression of several somatosensory molecular markers between human and mouse DRG tissue slices using dual-fluorescence *in situ* hybridization and immunohistochemistry. We thereby take into consideration – similar to intersectional genetic approaches – that nociceptor subtypes can be more precisely described by analyzing the expression of two instead of just one molecular marker. With this in mind we placed here a particular emphasis on the characterization of peptidergic (TRKA positive) nociceptive neurons. We decided to relate all chosen markers to *TrkA* expression for several reasons: TRKA, the high affinity nerve growth factor (NGF) receptor, is crucial for the proper development of most sensory neurons. At E13 roughly 80% of sensory neurons express TRKA (Ernsberger, 2009) and absence of either the receptor or its ligand in respective mouse models results in loss of 70–80% of DRG neurons (Crowley et al., 1994, Smeyne et al., 1994). In adult mice TRKA expressing nociceptors represent one of the largest sensory neuron subtypes with 40% of the DRG neurons being TRKA positive (Snider, 1994).

We selected the investigated markers based on two deliberations: first of all, we aimed to compare the presence and distribution of the main sensory neuron subtypes in both species and therefore investigated the expression of the neurotrophin receptors *TrkA* (*Ntrk1*), *TrkB* (*Ntrk2*) and *TrkC* (*Ntrk3*). These receptors can be used to categorize sensory neurons into three main cellular populations, nociceptors (Chen et al., 2006), mechanoreceptors (Perez-Pinera et al., 2008) and proprioceptors (Snider, 1994), based on the presence of TRKA, TRKB and TRKC, respectively. By including the receptor tyrosine kinase RET, which is expressed not only in some of the aforementioned subtypes but also in a substantial population of TRKA negative nociceptors (referred to as non-peptidergic nociceptors), we are covering essentially all main subtypes of sensory neurons. Recent studies (Usoskin et al., 2015, Li et al., 2016) using transcriptome analysis of single sensory neurons have shown that these main subtypes can be even further divided and specified. Future studies will reveal whether similar, more refined sub-categories of sensory neurons also exist in humans and what their specific functions might be. Secondly, we focused on ion channels that have been shown to be critically important in mediating nociception, such as the transient receptor cation channel TRPV1 and several voltage gated sodium channels (SCN8A–SCN11A, in this manuscript referred to as Na_v_ 1.6–Na_v_ 1.9).

By investigating the expression of our marker panel in reference to *TrkA*, we identified several major differences between human and mouse sensory tissue concerning the expression level, distribution and abundance of some of the analyzed markers. These results might have implications for translating data derived from mouse pain models to human patients.

## Experimental procedures

### Animals

Animal care and experimental setup was done according to the local animal welfare and their guidelines (Regierungspräsidium Karlsruhe, Germany).

8–11 weeks old female and male mice of a C57BL/6 background were used for the analysis. Mouse DRGs from L2 to L6 were freshly dissected, embedded in OCT (Sakura) and directly frozen on dry ice. 20 µm sections were cut on a Leica cryostat, air-dried and stored at −80 °C until further use.

### Human donors

Human DRG tissue was acquired from the Netherlands Brain Bank (NBB), a non-profit organization that collects human tissue. According to postmortem parameters (e.g. age, postmortem delay (time between death and isolation of the tissue) of 3–8 h or pH ≥ 6.3 (measured from cerebrospinal fluid)) human samples of non-diseased donors were chosen. Frozen tissue was shipped on dry ice and stored at −80 °C for at least 96 h.

For cryo-sections, human DRG tissue was acclimated at −20 °C for at least 1 h, embedded in OCT and directly frozen on dry ice. 20 µm sections were cut on a Leica cryostat, air-dried and stored at −80 °C until further use.

**Table:** 

DRG location	Sex/age	Cause of death
L4	F/77	Pulmonary metastasis of vulva carcinoma
L5	F/71	Renal insufficiency by hypertensive nephropathy
T6	M/51	Suicide by refusing food and water
T8	F/78	Cardiac insufficiency
T12	F/75	Myocardial infarct

L = lumbal; T = thoracal.

### *In situ* hybridization

*In situ* hybridization was carried out as previously described in Wende et al., 2012 and optimized for human tissue. In brief, thawed human and mouse cryo sections were fixed in 4% PFA for 30 min, acetylated and afterward permeabilized in 0.3% TritonX-100 in PBS. After pre-hybridization, hydrolyzed DIG- and/or FITC-labeled cRNA probes were added and hybridization was performed over night at 60 °C. The next day slices were washed twice with 2 × SSC/50% Formamide/0.1% N-Lauroylsarcosine at 60 °C and then treated with 20 µg/ml RNase A for 15 min at 37 °C. After washing twice in 2 × SSC/0.1% N-Lauroylsarcosine and twice in 0.2 × SSC/0.1% N-Lauroylsarcosine at 37 °C for 20 min each, sections were blocked in MABT/10% goat serum/1% blocking reagent.

For a monomeric, colorimetric NBT/BCIP staining, sections were incubated overnight with anti-DIG-AP-Fab (1:1000; Roche). After several washing steps with MAPT the color reaction was performed using NBT/BCIP.

For a double fluorescent *in situ* hybridization, sections were stained by two sequentially rounds of Thyramide signal amplification (TSA) steps with an intermediate peroxidase inactivation with 3% H_2_O_2_ for 2 h and 4% PFA for 30 min. Tissue was incubated with anti-FITC-POD (1:2000; Roche) or anti-DIG-POD (1:1000; Roche) over night at 4 °C. After intensive washing in MABT, the TSA reaction was performed by applying either Thyramide-Biotin on the third or Thyramide-Rhodamine on the fourth day for 30 min at room temperature. For the detection of Thyramide-Biotin a streptavidin-cy2 antibody (1:1000; dianova) was applied, whereas nuclei were stained with DAPI. Subsequently sections were washed and mounted with Immu-Mount (Thermo Fisher Scientific, Waltham, Massachusetts, USA).

*In situ* probes for mouse *Trpv1*, *TrkA* and *Na_v_1.6*–*1.9* were generated based on the sequences used by the Allen brain atlas. Mouse *TrkB*, *TrkC*, *Ret*, *Piezo2* as well as human probes for *PIEZO2* and *RET* were obtained from Dr. Hagen Wende (Institute of Pharmacology, University of Heidelberg). Full length clones for human *TRPV1* and *TRPA1* were provided by Dr. David Julius (University of California, San Francisco), and the generated *in situ* probes covered the complete open reading frame. The remaining *in situ* probes were amplified using the following primers and cloned into pBluescript SK(+).

**Table:** 

Gene	Fwd Primer (5′-3′)	Rev Primer (5′-3′)
*NTRK1 (TRKA)*	gacctcgagTCTGGAGCTCCGTGATCTGA	gacgcggccgcCCGTTGTTGACGTGGGTG
*NTRK2 (TRKB)*	attactcgagTGGAGCCTAACAGTGTAGATCCTGAGAAC	atatgcggccgcTGGTACTCCGTGTGATTGGTAACATG
*NTRK3 (TRKC)*	attactcgagTGGATGTCTCTCTTTGCCCAGC	atatgcggccgcATTCACCAGCGTCAAGTTGATGG
*SCN8A (Na_v_1.6)*	gacctcgagTCGCAAGCAGGAGGAGGTATCT	attagcggccgcCACCTGCCCAAGCATTAGA
*SCN9A (Na_v_1.7)*	attactcgagAAGGTGGGCAGCATTAGCAGAT	attagcggccgcTTGCCAAACACGGGATTGT
*SCN10A (Na_v_1.8)*	atatctcgagTGGATTCTCTGAAGGCAA	atatgcggccgcATCTGCAATGGGAAAGAGTT
*SCN11A (Na_v_1.9)*	attactcgaGCCTAGATAGTATGAAAGCAATGA	attagcggccgcACAGTCCTTCCTGGTGTCTTC

### Immunohistochemistry

Human and mouse DRG sections of 20 µm thickness were washed with PBS, fixed with 4% PFA for 30 min and blocked with 10% goat serum/0.1% TritonX-100 in PBS for 1 h at room temperature.

Primary antibodies (mouse ant-Tuj1 from Covance, 1:750; chicken anti-NF200 from Millipore, 1:25,000; mouse anti-NF200 (clone N52) from Sigma, 1:600 and rabbit anti-Brn-3a from Merck, 1:200) were diluted in 3% goat serum/0.1% TritonX-100 in PBS and applied over night at 4 °C. After rinsing 3 times with 0.1%Triton/PBS, sections were incubated with secondary antibodies (Alexa dyes 488 and 555 from dianova) and DAPI (diluted in 3% goat serum/0.1% TritonX-100) for 3 h at room temperature. Sections were washed with 0.1% TritonX-100/PBS and PBS and mounted with ImmuMount.

### Image acquisition and quantification analysis

Fluorescent picture acquisition was done on a Nikon A1R or C2 point scanning confocal microscope; bright field images were taken on a Ni-E wide field microscope (Nikon Imaging Center, University of Heidelberg). Images were analyzed and assembled using NIS-Element AR (Nikon), ImageJ/Fiji, Adobe Photoshop and Illustrator software.

For quantification analysis of each marker gene, positive neurons from 3 to 10 DRG sections per mouse (3–4 mice in total) and 3 sections per human DRG (3 human donors in total) were counted manually using Adobe Photoshop software. Averages from all sections were taken and percentages of double positive cells calculated.

### Area distribution analysis

For analyzing soma area distribution of *TrkA*, *TrkB* and *TrkC* positive cells, 3 DRG slices of 3 different donors were randomly chosen for each marker gene and analyzed using ImageJ/Fiji software. Regions of interest (ROI) were first set automatically by analyzing particles according to their pixel size in a thresholded, “binary”, black and white image. All pixels with values below a defined threshold were converted to white and pixels with values above a defined threshold were converted to black and included into the analysis. ROIs were also manually checked to refine automatically selected ROIs. Values were first measured and indicated in pixels and then converted in µm^2^ for displaying cell soma area. GraphPad Prism software was used for plotting the data.

### Signal intensity analysis

Signal intensity comparison of *TrkA* or *Ret* positive cells was analyzed in 5 randomly chosen DRG slices from mouse and human tissue, respectively, by using ImageJ/Fiji software. ROIs for both *TrkA* and *Ret* were set, by analyzing particles (as described for the area distribution analysis), and values were measured according to their mean grey value. For normalization of each slice, single mean grey values for *TrkA* or *Ret* were divided by the maximum mean grey value of each slice and multiplied by 100. GraphPad Prism software was used for plotting all *TrkA* and *Ret* ratios in percent.

### Statistical analysis

All quantifications are shown as mean ± standard error of the mean (SEM) of 3 unrelated human beings and 3–4 different adult mice. For statistical analysis, fractions of double-positive cells in individual slices were determined. The dependence of fractions within slices from a certain subject (single human being or mouse) was taken into account by applying a linear, mixed-effects model of the relationship between the fraction of double-positive cells and species as fixed effects. An additive random effect for each subject’s fraction level was introduced as random intercept. To this end, the software R (version 3.3.2) was used, with the lme function from the package nlme (version 3.1–131). The model was expressed in R as: lme (Fraction ∼ Species, random = ∼1|Subject) using default restricted maximum likelihood criterion. Residual plots provided no obvious discrepancies from homoscedasticity or normality.

## Results

### Expression of neurotrophin receptors

Somatosensory neurons in the dorsal root ganglion (DRG) are a heterogeneous population of cells that can detect and transduce a variety of different sensory stimuli. The different DRG neurons have unique molecular compositions and among the oldest and best characterized molecular markers are the neurotrophin receptors TRKA, TRKB and TRKC. Several studies characterizing adult DRGs in mice, rats and chicken have observed an almost mutually exclusive expression of TRKA, TRKB or TRKC in different subpopulations of sensory neurons (Snider, 1994, Hallbook et al., 1995, Chen et al., 2006, Perez-Pinera et al., 2008, Ernsberger, 2009). These observations resulted in the classification of TRKA positive sensory neurons as peptidergic nociceptors (Averill et al., 1995), responding to painful stimuli such as heat or noxious mechanical forces, while TRKB expressing sensory neurons mostly belong to the group of low threshold mechanoreceptors (LTMRs) responding to innocuous mechanical forces (Perez-Pinera et al., 2008) and TRKC positive sensory neurons are classified as proprioceptors, detecting muscle spindle tension (Snider, 1994). Correlating with the expression of one of the three neurotrophin receptor subtypes is a particular size range of the particular sensory neuron subtype: TRKA positive nociceptors showed a comparatively small diameter, while TRKB expressing LTMRs were found to be larger than TRKA neurons and TRKC positive proprioceptors – on average – outperform both other populations in size (McMahon et al., 1994).

**Fig. 1:**
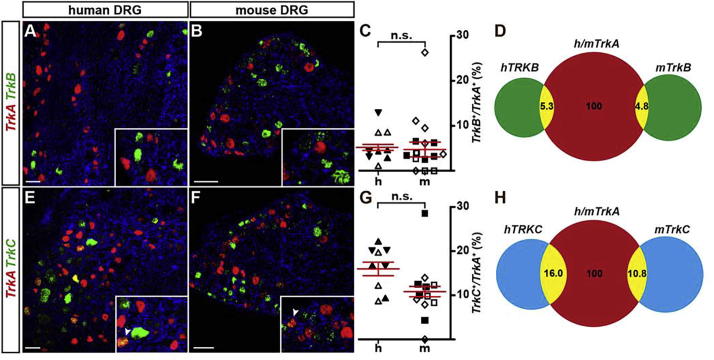
Comparison of *TrkB/TrkA* and *TrkC/TrkA* double positive sensory neurons in human and mouse dorsal root ganglia (DRG). (A, B, E, F) Representative dual-color fluorescence *in situ* hybridizations using probes to detect *TrkA* (red) and *TrkB* (green; A, B) or *TrkA* (red) and *TrkC* (green; E, F) were performed using human (A, E) or mouse (B, F) DRG tissue sections. Inserts show a close-up of labeled neurons with arrowheads in (E) and (F) indicating double-positive cells. (C, G) Quantifications of neurons double-positive for *TrkA* and *TrkB* (C) or *TrkA* and *TrkC* (G) are represented as scatter blots (mean ± SEM is shown). No significant (ns) difference was found when comparing tissues of both species (linear mixed model; p (*TrkB*) = 0.956; p (*TrkC*) = 0.111). Differently oriented or shaded triangles indicate percentages found in DRG sections obtained from different human individuals (*N* = 3), differently oriented or shaded squares depict percentages found in sections obtained from different mice (*N* = 3); (D, H) Venn diagrams depicting the percentage of *TrkB/TrkA* (D) or *TrkC/TrkA* (H) double positive cells from all *TrkA* expressing cells, in human and mouse tissue. Scale bar = 100 μm.

In order to investigate whether neurotrophin receptors are largely expressed in a mutually exclusive fashion also in adult human sensory neurons and to ascertain if the size distribution of hDRG neurons is also shifted in ascending order with the expression of *TRKA*, *TRKB*, or *TRKC* respectively, we performed dual-color fluorescence *in situ* hybridizations and compared the fraction of cells expressing both, *TrkA* and *TrkB* or *TrkA* and *TrkC* in human versus mouse DRGs. *TRKB/TRKA* double-positive neurons in human DRGs (Fig.1A–C) showed that 5.3 ± 0.7% of *TRKA* positive neurons also express *TRKB*, similar to 4.8 ± 1.7% of *TrkB/TrkA* positive neurons in mice (Fig.1A–C). The percentage of *TRKA* positive neurons also expressing *TRKC* in human DRGs was 16.0 ± 1.5%, similar to 10.8 ± 1.2% in mouse DRGs (Fig.1D–F). The fractions deduced from human DRGs did not significantly differ to fractions deduced from mouse sensory tissues (*TrkB/TrkA*, *p* = 0.956; *TrkC/TrkA*, *p* = 0.111; linear mixed model). We therefore concluded that, similar to mice, only small groups of human DRG neurons express *TRKA* together with either *TRKB* or *TRKC*.

In addition, also the relative total number of DRG neurons expressing *TrkB* or *TrkC* compared to the relative number of DRG neurons expressing only *TrkA* was similar in human and mouse DRGs (human *TRKB*: 27.8 ± 7.3%, mouse *TrkB*: 36.2 ± 13.5%, *p* = 0.531, linear mixed model; human *TRKC*: 39.5 ± 9.9%, mouse *TrkC*: 53.8 ± 5.5%, *p* = 0.295, linear mixed model; Fig.1D, H).

**Fig. 2:**
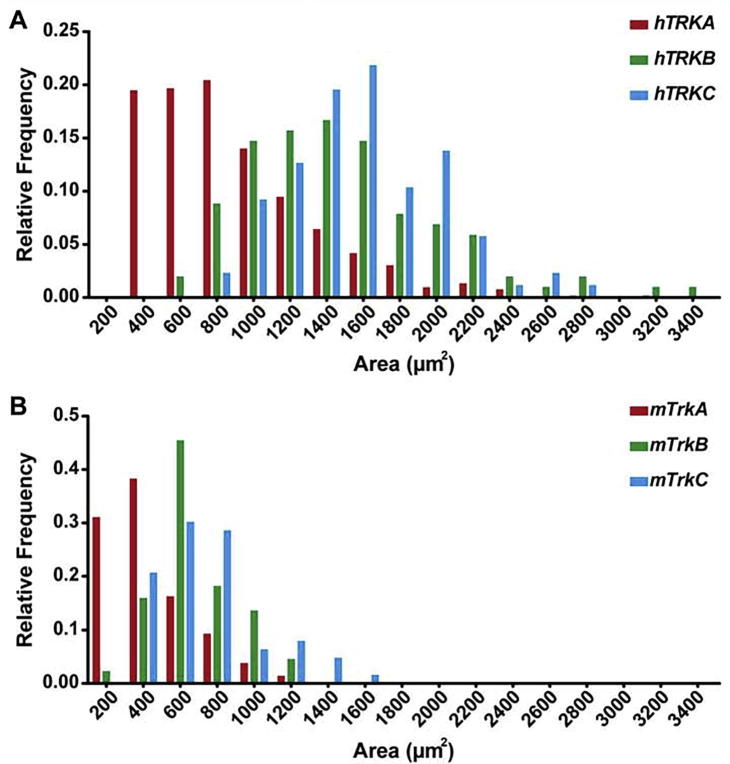
Comparison of the size distribution of *TrkA*, *TrkB* and *TrkC* positive cells in human versus mouse DRGs. (A) Distribution of the cell size (area in μm^2^) of *TRKA* (red), *TRKB* (green) and *TRKC* (blue) positive neurons in human DRGs. In human sensory tissue, the majority of *TRKA* positive cells exhibits a size around 300–700 μm^2^, while *TRKB* positive cells peak around 1200–1600 μm^2^ and *TRKC* positive cells around 1600 μm^2^. (B) Sensory neuron size distribution in mouse DRGs shows that the majority of *TrkA* positive cells are 100–300 μm^2^ in size, *TrkB* positive cells around 600 μm^2^ and *TrkC* positive cells around 600–800 μm^2^. By these measurements, human DRG neuron somata-areas are 1.5 to 3 times larger than mouse DRG somata when comparing the respective smallest and largest neurons of each TRK category.

To assess the size distribution of the different sensory neuron subtypes, we analyzed the cell area of *TRKA*, *TRKB* and *TRKC* positive human neurons (Fig.2A). Plotting the soma area for individual sensory neurons versus their relative frequency, we obtained a relative size distribution similar to that known for mouse DRG neurons (Fig.2B) with the majority of *TRKA* positive sensory neurons having a small area size (400–800 μm^2^), while *TRKB* positive neurons occupy a larger cell area (with a peak around 1400 μm^2^) and *TRKC* positive neurons being on average even larger (with a peak around 1600 μm^2^). When comparing the absolute cell sizes between mouse and human DRG neurons, we find – in agreement with previous literature (McMahon et al., 1994, Vega et al., 1994, Silos-Santiago et al., 1995, Molliver et al., 1997, Davidson et al., 2014) – that human sensory neurons are overall shifted to larger cell body sizes. Additionally, our analysis revealed that – on average – human DRG neuron somata-areas are 1.5 to 3 times larger than mouse DRG somata when comparing the respective smallest and largest neurons of each of the three TRK receptor categories (Fig.2A, B).

In summary, these comparative data show a rather similar distribution of double positive *TrkB/TrkA* and *TrkC/TrkA* sensory neuron fractions as well as a similar relative size distribution of mouse and human DRG neurons that correlates in ascending order with the expression of either *TrkA*, *TrkB* or *TrkC* neurotrophin receptors.

### Expression of *Trpv1* in peptidergic nociceptors

**Fig. 3:**
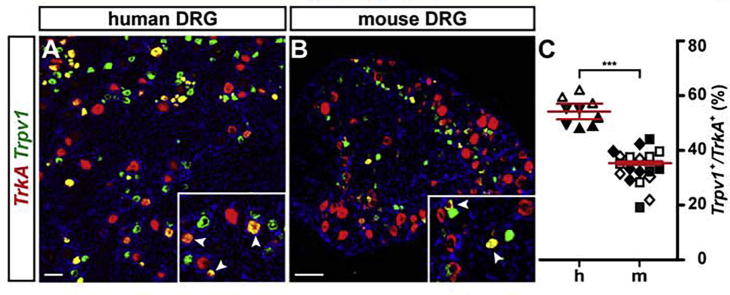
A higher percentage of *TRPV1/TRKA1* positive neurons reside in human compared to mouse DRGs. (A, B) Dual-color fluorescence *in situ* hybridizations using probes to detect *TrkA* (red) and *Trpv1* (green) in human (A) and mouse (B) DRG sections are shown. Inserts display close-ups with arrowheads indicating double-positive neurons. (C) Quantification of double-positive neurons in human (h) versus mouse (m) DRGs (expressed as percentage *Trpv1* positive cells of all *TrkA* positive cells), showed a significant difference between the two species (linear effects model; data shown as mean ± SEM; *p* = 0.0003 ***). Differently oriented or shaded triangles indicate percentages found in DRG sections obtained from different human individuals (*N* = 3), differently oriented or shaded squares depict percentages found in sections obtained from different mice (*N* = 4); scale bar = 100 μm. (For interpretation of the references to color in this figure legend, the reader is referred to the web version of this article.)

**Fig. 4:**
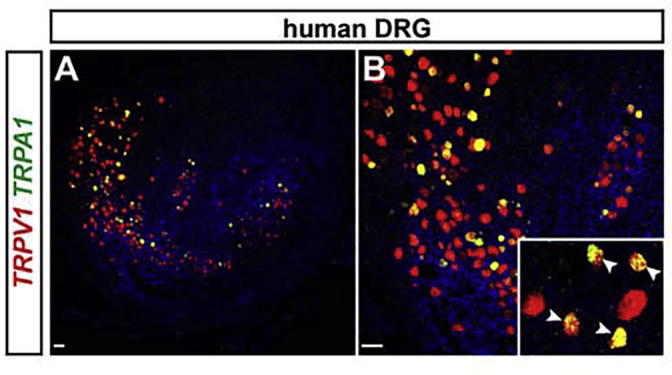
*TRPA1* is expressed in a subset of *TRPV1* positive neurons in human DRGs. (A, B) Representative dual-color fluorescence *in situ* hybridization detecting *TRPV1* (red) and *TRPA1* (green) transcripts in human DRG tissue sections. (A) Overview of a human DRG section and (B) zoom-in of (A). The insert in (B) shows a close-up with arrowheads depicting double-positive cells. Note that all *TRPA1* positive cells also express *TRPV1*. Scale bar = 100 μm.

Similar to classifying sensory neurons according to neurotrophin receptor expression, cationic Transient Receptor Potential (TRP−) ion channels have been used to divide sensory neurons in specific (functional) categories. The transient receptor channel V1 (TRPV1) is one of the most studied molecules of this group. TRPV1 was originally identified as the receptor for Capsaicin, the active component of chili peppers, and has been found to be a detector of noxious heat (>42 °C) (Caterina et al., 1997). While more recent results indicate that TRPV1 only plays a minor role in the acute detection of noxious heat (Yarmolinsky et al., 2016), it is well established that the channel is sensitized during inflammation and injury and therefore has emerged as a central target in the field of pain research (Carnevale and Rohacs, 2016). In the mouse TRPV1 is expressed in 22–38% of all sensory neurons (Ernsberger, 2008). The channel can be found in peptidergic as well as non-peptidergic nociceptors (Usoskin et al., 2015). Assessing the fraction of *Trpv1* positive sensory neurons among all *TrkA* positive peptidergic nociceptors by *in situ* hybridization, we find 35.4 ± 0.6% of *TrkA* positive neurons to express *Trpv1* in mouse DRGs compared to 54.2 ± 2.9% in human DRGs (Fig. 3). These data suggest that significantly more human peptidergic nociceptors express *TRPV1* than in the mouse (*p* < 0.001; linear mixed model). TRPA1, another member of the TRP channel family, which responds to pain-inducing chemical irritants such as mustard oil and that has been strongly implicated in pain and itch sensation (Story et al., 2003, Jordt et al., 2004, Kittaka and Tominaga, 2017), is known to be present in a subpopulation of TRPV1 positive sensory neurons in mice (Story et al., 2003). Our *in situ* hybridizations confirm these results and we find that in human DRGs essentially all *TRPA1* positive neurons also co-express *TRPV1* (Fig. 4).

### Presence of *RET* in human DRGs

During the development of the peripheral nervous system of the mouse, the neurotrophin receptors are expressed broadly and in an overlapping fashion. At E11–E11.5 70% to 75% of the developing sensory neurons express TRKC and/or TRKB. TRKA is even broader expressed and 80% of all neurons express TRKA at E13 (Ernsberger, 2009). TRK receptor expression becomes more refined at later stages of development and only at more mature postnatal stages TRKA, TRKB and TRKC are largely segregated in restricted subtypes of sensory neurons.

TRKA positive cells undergo a further transition during development and segregate into two groups: one subset that keeps expressing TRKA and one subset that downregulates TRKA expression and instead upregulates the tyrosine receptor kinase RET, the signaling receptor for the glial-derived neurotrophic factor, GDNF (Molliver et al., 1997). TRKA expressing neurons become peptidergic neurons, which express signaling mediators such as the peptides CGRP or Substance P. On the other hand the RET positive (and TRKA negative) population includes non-peptidergic neurons.

**Fig. 5:**
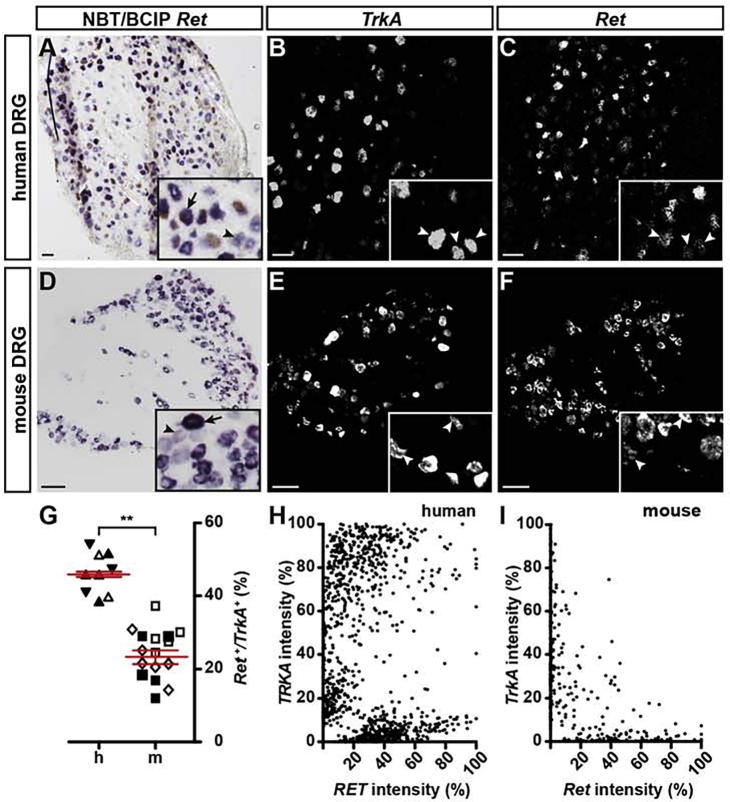
A neuron population expressing high levels of *TRKA* and medium to high levels of *RET* is present exclusively in human but not mouse DRGs. (A, D) Monochromatic images of single-color (NBT/BCIP) *in situ* hybridizations for *Ret* using human (A) and mouse (D) DRG tissue slices. Inserts show close-ups with arrows indicating neurons expressing high levels of *Ret* transcripts and arrowheads depicting neurons expressing low levels of *Ret*. (B, C, E, F) Monochromatic images of dual-color fluorescence *in situ* hybridizations for *TrkA* (B, E) and *Ret* (C, F) employing human (B, C) and mouse (E, F) DRG tissue sections. Inserts show magnifications of labeled neurons with arrowheads pointing to double-positive neurons. (G) Quantification of double-positive neurons (expressed as percentage of *Ret* positive cells of all *TrkA* positive cells) in human (h) versus mouse (m) DRG neurons, shows a significant difference in the amount of double-positive cells (linear mixed model, data shown as mean ± SEM; *p* = 0.002 **). Differently oriented or shaded triangles indicate percentages found in DRG sections obtained from different human individuals (*N* = 3), differently oriented or shaded squares depict percentages found in sections obtained from different mice (*N* = 3); (H, I) Analysis of the relative *TrkA* and *Ret* transcript expression levels (based on different fluorescent intensities) are shown for human (H) and mouse (I) DRG slices. Note the presence of a large group of *RET/TRKA* double-positive neurons in human DRGs (H) which is not detectable in mice DRGs (I). Scale bar = 100 μm. (For interpretation of the references to color in this figure legend, the reader is referred to the web version of this article.)

However, there are reports about the existence of a group of sensory neurons that conjointly express both markers, TRKA and RET, in an overlapping manner (Molliver et al., 1997, Golden et al., 2010, Stantcheva et al., 2016). These data show that about 10% of RET/TRKA double positive neurons exist in mouse DRGs. We analyzed whether *RET/TRKA* positive neurons are also found in human DRGs. Of all *TRKA* positive neurons identified by *in situ* hybridizations in human DRG neurons, we found 45.9 ± 0.7% (Fig.5A–C, G) to co-express *RET*, compared to 23.2 ± 1.8% in mice (Fig.5D–G). These data suggest that a *RET/TRKA* population of sensory neurons not only exists in human DRGs but that it is more extensive and proportionally almost double in size compared to mouse DRGs (*p* = 0.002 ∗∗; linear mixed model). In the mouse this sensory neuron population has been associated with the perception of itch (Usoskin et al., 2015, Stantcheva et al., 2016). Additionally, we also observed different levels of *RET* expression in human sensory neurons, similar to what has been observed for the mouse (Fig.5H, I). Analyzing the different expression levels by clustering *RET*, *TRKA* and *RET/TRKA* double positive cells according to their fluorescent *in situ* intensity levels, further confirmed the existence of several *TRKA* and *RET* populations in human DRGs. In particular, we found a considerable cohort of neurons to co-express high levels of *TRKA* and medium-to-high levels of *RET* in human DRGs that appears to be essentially absent from mouse DRGs (Fig.5H, I).

### Expression of *Piezo2* in *TrkA* positive nociceptors

We and others have recently shown, that the transmembrane protein PIEZO2 is an important mediator of mechanotransduction in RET and TRKB positive mechanoreceptors (Ranade et al., 2014, Schrenk-Siemens et al., 2015). While these LTMRs respond to innocuous mechanical stimuli, certain nociceptors do respond to noxious (painful) mechanical stimuli. Currently the molecular nature of mechanoreceptors in nociceptive neurons is unclear and it is unknown whether PIEZO2 is mediating mechano-nociception in a (sub-) fraction of pain-sensing neurons as well. This information is important because mechanical allodynia is a major health burden of chronic pain patients that is difficult to treat. While LTMRs play a critical role in mediating pathological forms of mechano-nociception (Campbell et al., 1988, Gracely et al., 1992, Sandkuhler, 2009, Dubin and Patapoutian, 2010), it is nevertheless important to investigate and learn whether PIEZO2 also is involved in mechanotransduction in nociceptive neurons.

**Fig. 6:**
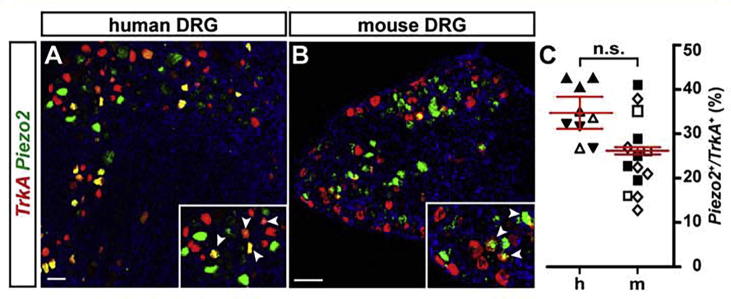
A subpopulation of *TrkA* positive neurons is expressing *Piezo2* transcripts in both human and mouse DRGs. (A, B) Double fluorescence *in situ* hybridizations of *TrkA* (red) and *Piezo2* (green) positive neurons in human (A) versus mouse (B) DRG sections. Inserts show magnifications with arrowheads indicating double-positive neurons. (C) Quantification of double-positive neurons in human (h) versus mouse (m) DRGs (expressed as percentage of *Piezo2* positive cells of all *TrkA* positive cells), shows no significant difference between the two species (linear mixed model; data shown as mean ± SEM; *p* = 0.059). Differently oriented or shaded triangles indicate percentages found in DRG sections obtained from different human individuals (*N* = 3), differently oriented or shaded squares depict percentages found in sections obtained from different mice (*N* = 3); scale bar = 100 μm. (For interpretation of the references to color in this figure legend, the reader is referred to the web version of this article.)

Previous studies have shown that *Piezo* transcripts can be found in mouse nociceptive neurons (Coste et al., 2010, Usoskin et al., 2015). However, the distribution of PIEZO2 has never been comprehensively analyzed in human DRG neurons. Therefore, we assessed the distribution of *Piezo2* expression in *TrkA* positive sensory neurons in mouse and human DRGs by *in situ* hybridization and found 26.2 ± 0.8% of *TrkA* positive nociceptors also expressing *Piezo2* in the mouse, compared to 34.8 ± 3.6% in the human DRG (Fig.6A–C). Although the human tissue showed a trend toward a larger fraction of *PIEZO2/TRKA* double positive cells, these data were not significantly different (*p* = 0.059; linear mixed model). These results suggest that PIEZO2 may indeed play a role in mechano-nociception in a subfraction of human peptidergic (and potentially also non-peptidergic) nociceptors.

### Presence of neurofilament heavy polypeptide in sensory neurons

The speed at which electrical information is transmitted throughout the nervous system depends to a large extent on (i) the insulation of the axon and (ii) its diameter. Schwann cells provide insulating myelination of peripheral nerves, while axon diameter depends on the type of sensory neuron itself (Wood et al., 1990). Neurofilaments have been correlated with the axon diameter of peripheral mouse neurons (Shelanski et al., 1994, Lariviere and Julien, 2004) and three subunits, light, medium and heavy, have been identified, based on their molecular weight. Neurofilament heavy polypeptide (NEFH) has been widely used as a molecular marker of fast-conducting so-called A-fibers. Two A-fiber types are typically distinguished: Aβ-fibers and Aδ-fibers. Both fiber types are myelinated but the Aβ-fibers, which are found in LTMRs, have a larger axon diameter and are conducting action potentials faster than Aδ-fibers. The latter are found to largely comprise nociceptors. The third class of fibers, so called C-fibers, are unmyelinated, display the slowest conduction velocity and are comprising the largest group of nociceptive neurons (Julius and Basbaum, 2001).

**Fig. 7:**
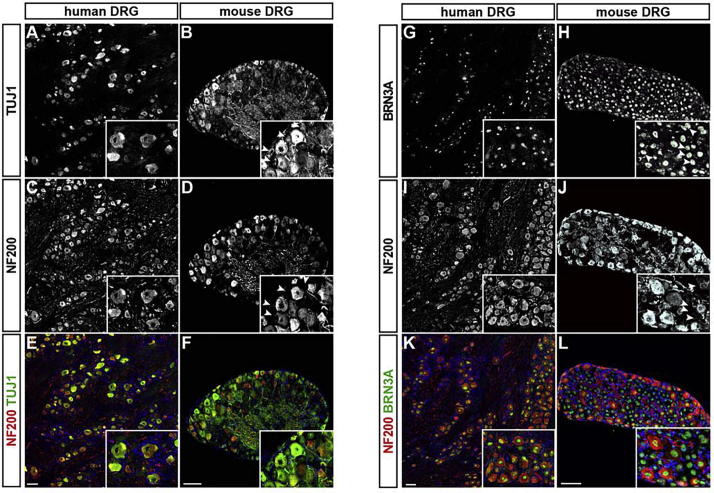
Most if not all human DRG neurons express the neurofilament heavy (NEFH; NF200) polypeptide. (A–L) Immunohistochemistry using antibodies to detect β-tubulin III (Tuj1), a pan-neuronal marker, BRN3A, a pan-sensory marker and neurofilament heavy (NF200) to label human and mouse DRG tissue sections. Two different NF200 antibodies have been used: (C–F) chicken anti-NF200 from Millipore and (I–L) mouse anti-NF200 (clone N52) from Sigma. Double fluorescence antibody labeling for Tuj1 (A, B, E and F) and NF200 (C–F) in human (A, C, E) and mouse (B, D, F) DRG sections and for BRN3A (G, H, K and L) and NF200 (I–L) in human (G, I, K) and mouse (H, J, L) DRG sections (Tuj1: green; NF200: red; E, F: BRN3A: green; NF200: red; K, L). Representative monochromatic images are shown in A–D and G–J and overlays are presented in E–F and K–L. Inserts show close-ups with arrowheads depicting mouse neurons being positive for Tuj1 (B) or BRN3A (H) but negative for NF200 (D) and (J). Note that in human DRGs almost no NF200 negative neurons were found. Scale bar = 100 μm. (For interpretation of the references to color in this figure legend, the reader is referred to the web version of this article.)

Since NEFH, has been widely used to characterize – and categorize – sensory neurons in rodent DRGs (Julius and Basbaum, 2001), we investigated whether this molecular marker is also useful to discriminate different sensory neuron subtypes in human DRGs. Using 2 different antibodies that recognize NEFH, we quantified the proportion of sensory neurons in human DRGs that express neurofilament heavy polypeptide and compared the result to numbers obtained from mouse DRGs. We co-labeled human and mouse DRG sections with antibodies recognizing the heavy subunit of neurofilament (NF200) together with either the pan-neuronal marker tubulin beta-III (TUBB3; clone Tuj1) or the pan-sensory marker POU4F1 (BRN3A). In the mouse only 61.7 ± 0.4% of all TUBB3 cells and 48.6 ± 0.4% of all BRN3A cells expressed NF200, whereas nearly all sensory neurons in the human DRG expressed this subunit (97.3 ± 1.2% co-expression for TUBB3 and 98.1 ± 0.4% co-expression for BRN3A; in both cases *p* < 0.001; linear mixed model; Fig.7A–L). While surprising, this result is in agreement with an older DRG study (Vega et al., 1994) and demonstrates that NEFH can be found in most, if not all human sensory neurons, independent of their respective subtype. These data also demonstrate that the heavy subunit of neurofilament is not a useful marker for categorizing human sensory neurons and points to further differences between the human and rodent somatosensory systems.

### Expression of sodium channels in *TrkA* positive sensory neurons

Different types of voltage-gated sodium channels (VGSCs) play crucial roles in transmitting pain signals to higher brain centers and a variety of pain-related disorders have been linked to mutations in sodium channels (Liu and Wood, 2011, Kanellopoulos and Matsuyama, 2016). In particular, function-compromising mutations in the genes encoding Na_v_1.7, Na_v_1.8 and Na_v_1.9 are causative to devastating pain disorders. As such, functional mutations in Na_v_1.7 cause congenital insensitivity to pain (CIP), a disease in which affected individuals can not feel any (physically inflicted) pain while other sensations, such as discrimination of different textures, are apparently not altered. Inherited primary erythromelalgia (IEM), paroxysmal extreme pain disorder (PEPD) and small fiber neuropathies (SFN) are also causally linked to functional mutations of the Na_v_1.7 locus (Kanellopoulos and Matsuyama, 2016). Pathological forms of pain can also be caused by mutations in Na_v_1.8 and Na_v_1.9 channels. Mutations in Na_v_1.8 can lead to SFN while Na_v_1.9 malfunction can cause a heritable form of pain insensitivity or familial episodic pain. The involvement of the different channels in pain related diseases in humans, rendered them important targets for the development of new analgesic drugs. However, the distribution of Na_v_ channels in the somatosensory system has not been fully elucidated and thus far drug discovery efforts did not yield any new therapies (Liu and Wood, 2011, Han et al., 2016, Kanellopoulos and Matsuyama, 2016). Additionally, discrepancies between Na_v_-related mouse and human pain phenotypes have been reported (Nassar et al., 2004, Weiss et al., 2011, Minett et al., 2012, Minett et al., 2014). Thus it is important to analyze Na_v_ channel expression patterns in human and mouse DRGs in order to understand similarities and differences of their respective somatosensory phenotypes.

**Fig. 8:**
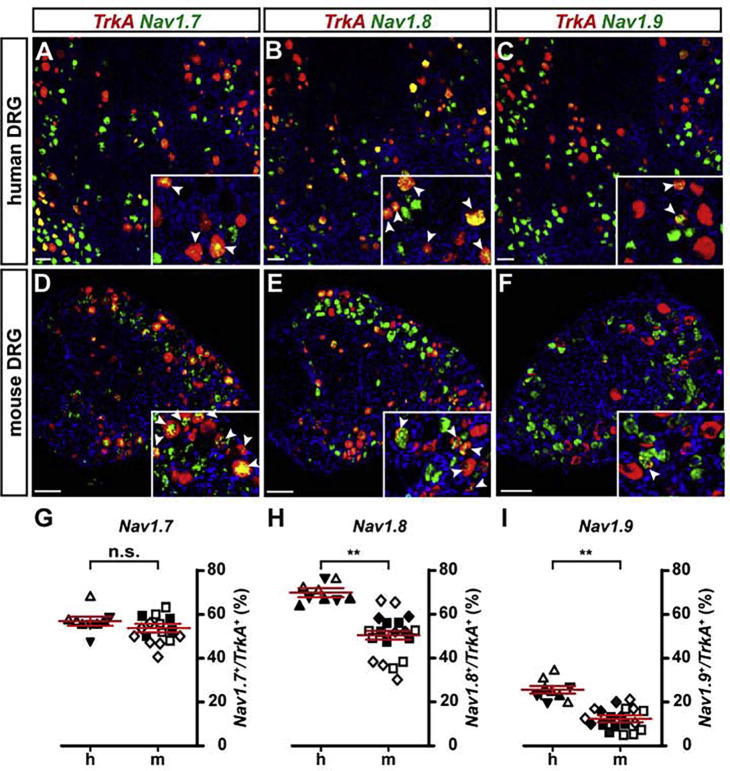
Analysis of Na_v_-channel expression in *TrkA* positive neurons of human and mouse DRGs (A–F) Dual-color fluorescence *in situ* hybridizations of *TrkA* (red) and Na_v_1.7 (green; A, D), Na_v_1.8 (green; B, E) and Na_v_1.9 (green; C, F) using human (A–C) and mouse (D–F) DRG tissue sections. Inserts show a close-up of labeled cells, arrowheads point to double-positive cells. (G–I) Quantification of double-positive neurons in human (h) versus mouse (m) DRGs (expressed as percentage of Na_v_ positive cells of all *TrkA* positive cells), showed no significant difference for Na_v_1.7 (G; *p* = 0.281) but significant differences were observed for Na_v_1.8 (H; *p* = 0.009 **) and Na_v_1.9 (I; *p* = 0.003 **; linear mixed model; data shown as mean ± SEM). Differently oriented or shaded triangles indicate percentages found in DRG sections obtained from different human individuals (*N* = 3), differently oriented or shaded squares depict percentages found in sections obtained from different mice (*N* ≥ 3); scale bar = 100 μm. (For interpretation of the references to color in this figure legend, the reader is referred to the web version of this article.)

We therefore investigated the distribution of Na_v_-channel expression in *TrkA* positive DRG neurons of both species using *in situ* hybridization. We found 42.9 ± 2.7% of *TrkA* positive neurons to co-express Na_v_1.6 in mice versus 35.2 ± 5.9% in human DRG neurons. These results were not significantly different (*p* = 0.204; linear mixed model). The fraction of *TrkA* positive neurons co-expressing Na_v_1.7 was found to be 53.8 ± 2.0% in the mouse versus 57.0 ± 2.1% in human DRGs (Fig.8A, D, G). Again, no significant difference was detected (*p* = 0.281; linear mixed model). In contrast, we found significant differences for the expression of Na_v_1.8 as well as Na_v_1.9: in the mouse DRGs we found 50.6 ± 2.0% of *TrkA* positive sensory neurons to co-express Na_v_1.8 compared to 69.8 ± 2.0% in human DRGs (*p* = 0.009; linear mixed model; Fig.8B, E, H). The difference in Na_v_1.9 expression was also significantly different between the two species, with 12.4 ± 1.6% of *TrkA* positive mouse neurons co-expressing the sodium channel, compared to 25.6 ± 1.7% in human sensory neurons (*p* = 0.003; linear mixed model; Fig.8C, F, I).

Collectively, these data demonstrate clear differences between human and mouse adult DRGs regarding the fractions of Na_v_1.8/*TrkA* and Na_v_1.9/*TrkA* double-positive sensory neurons, which may have functional consequences for the transmission of pain signals and/or the propagation of signals encoding other somatosensory modalities.

## Discussion

For decades, rodent animal models have served as invaluable tools to investigate and understand the molecular basis of diverse human diseases including pathological (and chronic) forms of pain (Mogil, 2009). It is without question that these models helped tremendously to broaden our basic knowledge concerning causes, onset and progression of debilitating forms of pain. Nevertheless, the development of new drugs and unconventional therapeutic options is lacking behind and has not met expectations. Especially the limited success of translating findings from rodent models to the human disease situation has raised doubts about the benefit chronic pain patients can expect from studies performed in mice and rats (Hill, 2000, Percie du Sert and Rice, 2014, Burma et al., 2016).

Studying pain in humans is difficult: pain is a very individual experience, and experimental pain research can only be performed to a very limited degree in human subjects. Thus, it is no matter of debate that refined model systems are needed. It is noteworthy that recent developments in human embryonic stem cell- or human induced pluripotent stem cell-technology may allow the generation of (patient-derived) sensory neurons and thereby provide a missing link to test the relevance of rodent-derived data for human patients. Future work is required to assess whether (and to what extent) such human nociceptor-like cells resemble their native counterparts and how reproducibly they can be generated.

In the meantime, rodents will continue to serve as important and necessary model organisms for pain research. Therefore, it is of critical importance to investigate similarities and differences between humans and rodents regarding cellular and molecular aspects of sensory neurobiology in order to gauge what aspects of this research have a translational potential and, additionally, to indicate differences that may explain shortcomings concerning therapeutic expectations.

The comparative study presented here provides fresh new insight into the differences and similarities of human and mouse sensory ganglia. To this end we have investigated the presence of a panel of selected marker molecules that have been described to be involved in nociception and assessed their distribution and co-expression in reference to the neurotrophic receptor TRKA, that is largely present in peptidergic nociceptors. To our knowledge, this is the first time that such a marker analysis has been done in this detail and the differences we found already at this – rather coarse – level emphasize the need for further, more detailed, comparative studies in the future.

The expression and specific distribution of neurotrophin receptors in sensory neurons during development and in the adult has been intensively studied in the past and was used as one criteria, to classify the neurons into mechanoreceptive, proprioceptive and nociceptive subtypes.

In order to ascertain whether these three major sensory neuron categories are also non-overlapping in human sensory ganglia, we investigated the co-expression of either *TrkB* or *TrkC* with *TrkA* by using *in situ* hybridizations. Our analysis revealed an overlap of two different *Trk* receptors only in a minority of cells and these results were highly similar in sensory ganglia obtained from both organisms. This suggests that, similar to what has been shown in rodent experiments (McMahon et al., 1994), TRK receptors – at least at the adult stage – largely serve non-redundant functions also in human DRGs.

The correlation of the soma size of sensory neurons with neurotrophin receptor expression offers one way of how to classify sensory cells into different categories: neurons with small diameter size mostly express TRKA, medium to large diameter neurons mostly express TRKB, and TRKC is found in a spectrum of sensory neurons that extents to even larger somata sizes. Analyzing the distribution of somata sizes as a function of *TRKA*, *TRKB* and *TRKC* expression in human tissue, we observed a highly similar relative size distribution across *TRKA*-, *TRKB*-, or *TRKC*-expressing human neurons compared to results found in the mouse. Not surprisingly, in terms of absolute size, the human cells are considerably larger than the mouse cells. Considering the distribution of neurotrophin receptors and the cell size as one way of characterizing the general composition of DRGs, we found no significant differences between human and mouse sensory ganglia at this level of our characterization.

Sensory neurons represent a very diverse group of neurons, and over the last decades scientists have characterized and sorted them into different categories based on (i) marker expression (Usoskin et al., 2015, Li et al., 2016) (ii) functionality (Basbaum et al., 2009) or (iii) target innervation (Marmigere and Ernfors, 2007). A recent unbiased approach by the research groups of P. Ernfors and X. Zhang, made use of single cell transcriptome analysis to classify sensory neuron subtypes by clustering their transcriptome. Usoskin et al. define 11 main sensory neuron subtypes in mouse DRGs. Again – and using such an orthogonal approach compared to *in situ* hybridization – almost all analyzed cells expressed only one of the three neurotrophin receptors. Another receptor important in sensory neuron development and function that thus far has been extensively studied in rodent sensory ganglia is the receptor tyrosine kinase RET (Ernsberger, 2008). Besides other molecules, it is the receptor for GDNF and is expressed in roughly 60% of all sensory neurons in mice or rats (Molliver et al., 1997, Bennett et al., 1998, Kashiba et al., 1998, Josephson et al., 2001, Zwick et al., 2002, Kashiba et al., 2003). In mice RET positive cells are generated in two developmental waves, with a first wave resulting in RET/TRKB double-positive, LTMRs and a second wave of late RET cells, forming the large heterogeneous population of non-peptidergic nociceptors (Marmigere and Ernfors, 2007). In addition, some smaller populations of RET positive cells, not fitting the two aforementioned criteria, have also been described: a RET/TH double-positive population of C-fiber low threshold mechanoreceptors (C-LTMRs) and a small population of RET/TRKA double positive cells. The latter population comprises around 10% of the entire RET population in mice (Molliver et al., 1997, Golden et al., 2010) and recent studies could show, that this population is involved in the sensation of itch (Usoskin et al., 2015, Stantcheva et al., 2016). Additionally, Stantcheva et al., could show that RET expression levels differ across sensory neurons, with neurons expressing high levels being most likely mechanoreceptors while some low expressers were found to be devoid of IB4 (a marker of non-peptidergic nociceptors) but co-express TRKA, TRPV1, Somatostatin and pruritogen receptors. The latter category has been suggested to constitute presumed itch-sensory neurons.

Investigating the fraction of *Ret/TrkA* double positive cells in our study, we found a significant difference between human and mouse tissue: in human DRGs almost double the fraction of *TRKA* positive cells co-express *RET* compared to mouse DRG cell counts. This finding is surprising, as it has been previously shown in several studies employing rodent animal models, that TRKA and RET co-expression is largely observed only during development but that TRKA expression is downregulated in those cells that instead upregulate RET expression (in the mouse this “TRKA-to-RET switch” begins around embryonic day E16 and is completed after birth). In rodents this event has been suggested as a hallmark for the emergence of non-peptidergic nociceptors (Molliver et al., 1997, Franck et al., 2011). Our data show that almost 50% of *TRKA* positive sensory neurons in human subjects continue to express *RET* mRNA throughout adulthood, making it a major sensory neuron subtype in humans. We can only speculate about the function of this population but a recent study by Stantcheva et al. linked the (rather small murine) population of *Ret/TrkA* double positive sensory neurons to itch sensation and demonstrated that they also express TRPV1(Stantcheva et al., 2016), which is consistent with a larger fraction of *TRPV1/TRKA* positive neurons that we find in human DRGs (Fig. 3).

Because we are exposed to numerous and divers (environmental) pruritogens, our need to protect ourselves might account evolutionary for the increased number of this particular sensory subtype in human beings. Further functional analysis of human DRG neurons would have to verify whether the *RET/TRKA* population in humans is indeed involved in itch sensation or whether only a sub fraction of them serve this function. In this regard it is noteworthy that, similar to data obtained from mouse DRGs (Stantcheva et al., 2016), we observed obvious differences in the expression level of *Ret* mRNA across sensory neurons. It is tempting to speculate that this allows further segregation of functionally discrete subtypes of human sensory neurons as has been demonstrated in the mouse.

We have also attempted to reveal the aforementioned important subset of sensory neurons labeled by the gene product for Tyrosine hydroxylase (TH), forming the group of C-LTMRs. We tried to reveal TH RNA and protein by different experimental means. However, for technical reasons that are not entirely clear to us – perhaps less-than-optimal quality of the remaining post-mortem human DRGs – we were not able to obtain any conclusive data on the expression of TH in human sensory neurons.

Beside its involvement in itch sensation, TRPV1 is classically known for its role as an integrator of various inflammatory and painful stimuli including noxious heat (>42 °C), lipid mediators, tissue acidosis and others. Additionally, numerous signaling mediators sensitize the ion channel as a consequence of tissue injury and inflammation. These properties have rendered TRPV1 an interesting analgesic drug target and several pharmaceutical companies have heavily invested in pharmacological agents that can inhibit TRPV1 (Moran et al., 2011, Nilius and Szallasi, 2014, Carnevale and Rohacs, 2016). Unfortunately, targeting the channel to alleviate pathological forms of pain, results in severe adverse effects, one of them being an increase of body temperature. Currently, these shortcomings have prevented the development of a breakthrough TRPV1 inhibitor that can be safely and systemically administered to pain patients.

TRPV1 is expressed in 22–38% of all sensory neurons in mice (Orozco et al., 2001, Zwick et al., 2002). At the protein/functional level, expression of the receptor is mainly found in peptidergic C-fiber nociceptors and, to a lesser extent, also in non-peptidergic nociceptors (Cavanaugh et al., 2011). This is somewhat in contrast to results obtained from single-cell sequencing experiments that show robust *Trpv1* expression in non-peptidergic neurons as well (Usoskin et al., 2015, Li et al., 2016). Investigating the fraction of *TrkA* positive neurons co-expressing *Trpv1*, we found a significant difference between mouse and human tissue: human sensory neurons do have a larger population of double-positive cells, compared to mice (54.2 ± 2.9% versus 35.4 ± 0.6% respectively; *p* < 0.001; linear mixed model).

Due to its role as detector of noxious stimuli, TRPV1 is important to avoid tissue damage owing, for example, to accidental burns. Similarly, thermal hyperalgesia caused by inflammatory induced sensitization of the TRPV1 channel, serves as a protective mechanism in order to prevent further damage of an already impaired body part. TRPV1/TRKA positive neurons have been associated with mediating responses to acute, highly-localized pain stimuli.

Further, we found a significant difference in human versus mouse sensory tissue, concerning the presence of the heavy neurofilament isoform, NEFH. Neurofilaments are part of the neuronal cytoskeleton and contribute to radial axonal growth, thereby influencing conduction velocity. NEFH, is found in the rodent peripheral nervous system in so called A-fibers, which comprise the Aβ- and Aδ-fibers. Aβ-fibers are classified as LTMRs and are responsible for transmitting information about innocuous mechanical stimuli, while Aδ-fibers belong to the group of nociceptors that mediate acute, well localized fast pain (Basbaum et al., 2009). Both are myelinated by Schwann cells and therefore transmit electrical impulses rather fast. Roughly 46% of sensory neurons in mice express *Nefh* (Li et al., 2016) on RNA level. On the protein level, we find in our study about 50–60% of NEFH positive neurons in mice. This is in stark contrast to NEFH protein expression in human sensory neurons where we find most if not all sensory neurons to express NEFH (97.3 ± 1.2%), using 2 different types of anti-NEFH antibodies. Our findings confirm previous data (Vega et al., 1994). One could speculate that the ubiquitous expression of NEFH in human sensory neurons correlates with an overall increase in conductance velocity in the respective fibers, a feature that could theoretically account for the larger body size of humans. But several studies have shown no major differences between the conductance velocities in humans versus mouse or rat, using microneurographic techniques (Handwerker et al., 1991, Cain et al., 2001, Hagbarth, 2002). We can therefore only conclude, that NEFH labeling in human sensory neurons is unsuitable to classify specific sensory subtypes and its expression appears not to correlate with increased fiber-type specific conduction velocities.

Cutaneous pain signals are largely transmitted via C-fibers, which consist of both: mechano-sensitive and mechano-insensitive fibers. While in the past years the large transmembrane protein PIEZO2 has been found to be the main transducer of innocuous mechanical stimuli mediated by LTMRs, it is still not resolved what molecules are involved in noxious mechanical sensation within nociceptors (Ranade et al., 2014, Schrenk-Siemens et al., 2015). PIEZO2 is still a possible candidate as its role in mechano-nociception has not unequivocally been ruled out. A prerequisite for its involvement is its presence in the respective cell types. Our *in situ* hybridization experiments show the presence of *Piezo2* in *TrkA* positive nociceptors in mice and men: 26% of *TrkA* neurons also express *Piezo2* in mice, compared to 35% in humans. Although there is a tendency of human sensory neurons to display a larger population of *TRKA* neurons co-expressing *PIEZO2*, the difference between the two species was not significant. We can therefore draw the conclusion that *Piezo2* is present in both species in a considerable fraction of *TrkA* positive cells and thus could be involved in mechano-nociception. But further studies are needed to prove this.

In the past decade, VGSCs have been recognized as being heavily involved in the generation and transmission of pain signals. Three of the nine homologous members of the VGSC family – Na_v_1.7, Na_v_1.8 and Na_v_1.9 – are important for nociceptive-signal transmission. All three members have also been implicated in heritable pain disorders such as CIP, IEM, PEPD and small fiber neuropathy (Kanellopoulos and Matsuyama, 2016).

In our study, we find no difference regarding the distribution of Na_v_1.7/*TrkA* double positive sensory neurons between mouse and man, but we did find a significant higher fraction of *Na_v_1.8/TRKA* positive neurons in humans (69.8 ± 2.0%) versus mouse (50.6 ± 2.0%) as well as *Na_v_1.9/TRKA* positive neurons (humans 25.6 ± 1.7% versus mouse 12.4 ± 1.6%).

According to a recent study, the electrical properties of human Na_v_1.8 appears to differ from its rodent orthologous and the human channel shows slower inactivation kinetics, larger persistent and ramp currents, longer-lasting action potentials resulting in increased firing frequency of the neurons (Han et al., 2015). Theses observed differences may be of relevance when discerning discrepancies of pain pathologies between Na_v_1.8 mouse mutants and human Na_v_1.8 channelopathies.

## Conclusions

The present comparative study provides for the first time a detailed, expression profile – at cellular resolution – of several marker molecules that are expressed in human and mouse *TrkA*-positive sensory neuron populations. The identified molecular differences may guide future efforts to resolve physiological (and pathological) differences between the human and the rodent pain pathways. This study may also be a useful reference – particularly if extended to include non-peptidergic (TRKA negative) sensory neurons and further molecular markers – for the comparison and characterization of hESC- and hiPSC-derived human-like nociceptors.
